# Intention to give birth in the health institutions and associated factors among women who gave birth in the last 6 months in Debre Berhan town, North Showa zone, Ethiopia: A community-based cross-sectional study

**DOI:** 10.3389/fmed.2022.917678

**Published:** 2022-11-16

**Authors:** Nakachew Sewnet Amare, Abebayehu Melesew Mekuriyaw, Getaye Worku Tesema, Yeshinat Lakew Ambaw

**Affiliations:** School of Nursing and Midwifery, Asrat Woldeyes Health Science Campus, Debre Berhan University, Debre Berhan, Ethiopia

**Keywords:** childbirth, Ethiopia, health institution, intention, labor and delivery

## Abstract

**Background:**

Institutional delivery is a proxy for skilled birth attendance, which is an important intervention to reduce maternal and neonatal mortality. Even though institutional delivery has such importance, significant numbers of women in Ethiopia do not prefer to give birth in health institutions. This study aimed to assess women’s intention to give birth in health institutions and associated factors among women who gave birth in the last 6 months in Debre Berhan town, North Showa Zone, Ethiopia, 2020.

**Materials and methods:**

A community-based cross-sectional study was conducted among women who gave birth in the last 6 months in Debre Berhan town from October 30 to November 30, 2020. A cluster sampling technique was used to select study participants. Pretested semi-structured interviewer-administered questionnaires were administered. A logistic regression model was performed, and adjusted odds ratios with a 95% confidence interval based on *p* < 0.05 were used to identify statistically significant variables.

**Result:**

This study found that a total of 689 (88.8%) (95% CI: 86.6, 91%) respondents intended to deliver in the health facility. Being multiparous [AOR = 0.18 (95% CI: 0.08, 0.36)], having planned pregnancy [AOR = 3.1 (95% CI: 1.6, 5.9)], had no complications during previous delivery (AOR = 6.0 (95% CI: 3.5, 10.4)], and received respectful maternity care (RMC) during preceding delivery [AOD = 1.8 (95% CI: 1.05, 3.10)] are significantly associated with women’s intention to give birth in the health institution.

**Conclusion:**

Childbirth is a special event that requires the safest place to save the lives of both the mother and newborn. In this study, the number of women who do not have the intention to give birth in the health institution is still high. Strategies to promote planned pregnancy, reduce complications during childbirth and provide RMC during childbirth should be designed and interventions should be implemented for all childbearing women.

## Background

Institutional delivery is giving birth in a place where medical facilities and skilled birth attendants are available. Giving birth in a health institution is one of the important practices to improve maternal health status and wellbeing and to reduce maternal mortality through providing safe delivery services with reducing complications related to and occurring during childbirth (1, 2).

Over 300,000 women die globally each year, and this huge maternal death is a global public health concern (3). These deaths mainly occur during the time of labor and delivery and the first 24 h after delivery. Using health institutions for childbirth is, therefore, playing an important role in reducing maternal deaths and is helpful to attain the 2,030 sustainable development goals (SDGs) of improving maternal health (4).

According to the World Health Organization (WHO) report, the utilization of skilled birth attendants in developing countries is very low, and only approximately 50% of pregnant women are delivered in health institutions by skilled birth attendants (5).

Although there has been a reduction in maternal and child deaths in Ethiopia since 2000, the maternal mortality and child mortality rates are still high, 412 per 100,000 live births and 67 per 1,000 live births, respectively (6).

Women’s intent for at-home deliveries is one of the reasons for the low utilization of health facility deliveries in developing countries, including Ethiopia (7, 8). This women’s intention is because of the satisfaction resulting from home delivery in contrast with health institution delivery. This intention toward facility delivery is based on previous experience or perceptions surrounding these services and other sociodemographic, obstetric, and reproductive health service-related factors (9). Therefore, this study intended to identify those factors associated with women’s intention to use health institutions for childbirth.

Even though providing respectful maternity care (RMC) could be important in improving women’s intention to give birth in a health institution, studies did not show an association between RMC and intention to give birth in a health institution. However, this study considered RMC during childbirth as one of the independent factors and tried to examine its association with the intention of women to use health facilities for childbirth. Since most studies done previously on this issue were institutional-based studies, conducting a community-based study is better for the representativeness of the findings to our target population in the area.

Determining the magnitude of women’s intention to give birth in the health institution and identifying the possible factors is important to design strategies and implement interventions. Therefore, this study aimed to assess the intention to give birth in the health institution and associated factors among women who gave birth in the last 6 months in Debre Berhan town, North Showa zone, Ethiopia.

## Materials and methods

### Participants and procedure

A community-based cross-sectional study was conducted in Debre Berhan town, North Showa zone, Ethiopia, from October 30 to November 30, 2020. The source populations for the study were all women who gave birth in Debre Berhan town, North Showa zone, Ethiopia. The study populations were all women who gave birth in the last 6 months during the data collection period, in Debre Berhan town, North Showa zone, Ethiopia. All women who gave birth in the last 6 months during the data collection period, in Debre Berhan town were included in the study. And women who are unable to communicate effectively due to serious illness were supposed to be excluded from the study. The sample size was calculated using the Epi Info Stat Calc version 7.2.1 population survey by taking the 95% confidence interval, the magnitude of the intention to use institutional delivery service and its predictors among pregnant women was 73.4% (10), the confidence limit (margin of error) was 5%, and a 10% non-response rate was added. The final sample size was calculated as follows:


$$n=\frac{{\left(z\alpha /2\right)}^{2}(p\left(1-p\right)}{d^{2}}=\frac{{\left(1.96\right)}^{2}(0.734\left(1-0.734\right)}{{\left(0.05\right)}^{2}}=300$$

where *z*α/2 is the *z* value for the 95% confidence level.

***d*** is the margin of error and

***p*** is proportion


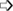
 by adding a non-response rate of 10% with two design effects (300 + 30) 2 = our final sample size was 660.

Debre Berhan town has a total of 9 kebeles, 6 kebeles were selected randomly, and by using a cluster sampling technique, we took all mothers in the selected kebeles who were eligible for the study. Finally, we found 776 eligible study participants, and all of them were included in our study.

### Data collection tools procedures

Data were collected using a structured and semi-structured interviewer-administered questionnaire with two parts. The first part contained baseline data regarding sociodemographic characteristics. The remaining part of the questionnaire comprised obstetrics-related questions and the question to know the women’s intent to give birth in health institutions. The questionnaires were developed in English by reviewing various literature, translated into the local language Amharic and then retranslated back to English to check the consistency. The data collection tool was reliable with a Cronbach’s alpha value of 0.79 and it is valid in the Ethiopia context. The data collection was undertaken for 1 month from October 30 to November 30/2020 by 12 trained midwife data collectors and four MSc midwife supervisors.

### Data quality control

To assure the data quality, training was given to data collectors and supervisors regarding the objectives of the study, data collection method, and significance of the study. During data collection, each data collector was supervised for any difficulties, and direction and necessary corrections were provided. The collected questionnaires were checked for completeness and on-spot corrective measures were taken both by data collectors and supervisors. A daily meeting was conducted among data collectors, supervisors, and the principal investigator for discussion regarding presenting difficulties and to assess the progress of data collection.

### Statistical analysis

The data were entered into EPI INFO™ 7 and exported to SPSS version 21 statistical software for analysis. Frequencies and cross-tabulations were used for descriptive statistics of the collected data, and the data are presented in tables. A binary logistic regression model was used to identify factors associated with women’s intention to use health institutions for childbirth. Those variables with a *p*-value less than or equal to 0.2 from the bivariable analysis were candidates for multivariable analysis. Variables with a *p*-value of less than 0.05 in multivariable analysis were declared statistically significant factors with the outcome variable.

### Operational definitions

#### Intention to give birth in a health institution

It was measured by asking women about their intention to use a health institution for the next delivery. The women were asked to choose either “Yes” or “No” (11).

## Result

### Sociodemographic characteristics of the participants

We found 776 postpartum women who were eligible to be a participant in the study, and all of them were interviewed with a 100% response rate. The mean age of the participants was 27.65 5.2 years, ranging from 19 to 45 years. The participants’ average family income was 6476.1, and the minimum and maximum family income were 200 and 80,000 Ethiopian birr (ETB), respectively. The majority, 731 (94.2%), of the participants were married. Regarding the educational status of the participants and their husbands, 22.8 and 18%, respectively, could not read and write (Table 1).

**TABLE 1:** Sociodemographic characteristics.

Characteristics	Frequency	Percent (%)
**Religion**		
Orthodox	634	81.7
Muslim	108	13.9
Protestant	32	4.1
Catholic	2	0.3
**Marital status**		
Single	31	4.0
Married	731	94.2
Divorced	8	1.0
Separated	6	0.8
**Educational status**		
Can’t read and write	178	22.8
Can read and write	98	12.6
Primary school	138	17.8
Secondary school	157	20.2
Diploma and above	295	26.4
**Husband educational status**		
Can’t read and write	140	18.0
Can read and write	124	16.0
Primary school	129	16.6
Secondary school	148	19.1
Diploma and above	235	30.3
**Ethnicity**		
Amhara	722	93.0
Oromo	48	6.2
Tigre	6	0.8
**Residence**		
Urban	479	61.7
Rural	297	38.7
**Occupation**		
Housewife	391	50.4
Private employer	66	8.5
Government employee	157	22.6
Merchant	73	9.4
Student	25	3.2
Daily labor	16	2.1
Farmer	30	3.9
**Husband occupational status**		
Farmer	288	37.1
Government employee	216	27.8
Merchant	160	20.6
Private employer	52	6.7
Student	8	1.0
Daily labor	28	3.6
Other*	24	3.1

*Includes driver (18), jobseeker (3), tailor (3).

### Obstetric/Health care service-related characteristics of the participants

Of the study participants, 508 (65.5%) were multiparous, and 678 (87.4%), and 720 (92.8%) of the participants had planned and wanted pregnancy, respectively. Among the women who had antenatal care (ANC) follow-ups, 325 (45.5%) had ANC follow-ups of approximately four and above. Among women who had ANC follow-up, the majority, 605 (84.6%) had ANC follow-up at the health center, and the remaining 139 (19.4%) and 89 (12.4%) had ANC follow-ups at the government hospital and private health institution, respectively. The majority, 684 (88.1%), of the participants’ labor was established spontaneously, and the majority, 502 (64.2%), of women’s duration of labor was less than 12 h. On the other hand, three-fourths of the participants stayed for more than 12 h in the health facility. Nearly half and three-fourths of the health care providers were females and midwives, respectively. Of the respondents, 422 (54.4%), 733 (94.5%), 613 (79.0%), and 423 (54.5%) were delivered at night, and the neonate was alive, had no complications during labor and delivery, and had delivered in the health center, respectively (Table 2).

**TABLE 2:** The obstetric/Health care service-related characteristics.

Characteristics	Frequency	Percent (%)
**Parity**		
Primi parous	268	34.5
Multiparous	508	65.5
**Planned pregnancy**		
Yes	678	87.4
No	98	12.6
**Wanted pregnancy**		
Yes	720	92.8
No	56	7.2
**Have ANC follow up**		
Yes	714	92.0
No	62	8.0
**Labor start**		
Spontaneous	684	88.1
Induced	68	8.8
C/s	24	3.1
**Mode of delivery**		
SVD	569	73.3
Instrumental	59	7.6
C/S	148	19.1
**Time in labor**		
Less than 12 h	502	64.7
Greater than 12 h	274	35.3
**Time stays in a health facility**		
Less than 12 h	249	32.1
12 to 24 h	291	37.5
>24 h	236	30.4
**Sex of health provider attending labor**		
Female	357	46
Male	419	54.
**The profession of health provider**		
Midwifery	574	74
Nurse	5	0.6
General practitioner	1	0.1
Intern	29	3.7
ISO	94	12.1
Gynecology	73	9.4
**Time of delivery**		
Day	354	45.6
Night	422	54.4
**Neonate alive**		
Yes	733	94.5
No	43	5.5
**Any complication during labor and delivery**		
Yes	163	21.0
No	613	79.0
**Place of delivery**		
Health center	423	54.5
Hospital	353	45.5

### Women’s intention to give birth in health institutions

Of all the participants, 689 (88.8%) (95% CI: 86.6, 91%) of the respondents had the intention to deliver in the health facility in their future pregnancy, and the rest 87 (11.2%) of the participants had no intention to deliver in the health facility in the future. The major reason for not being intended to deliver in the health facility was reported as not being satisfied with the service they received (37.9%).

### Factors associated with the intention to deliver in the health facility

By applying a binary logistic regression model and a multivariate analysis, this study identified variables, parity, having planned pregnancy, complications during labor and delivery, and having RMC, that showed significant associations with women’s intention to give birth in health institutions.

Those participants who were multiparous were 82% less likely to prefer to deliver in the health facility in the future compared to those who were prim parous [AOR = 0.18 (95% CI: 0.08, 0.36)]. Those women who had planned pregnancies were 3.1 times more likely to have the intention to deliver in the health facility than those who had unplanned pregnancies [AOR = 3.1 (95% CI: 1.6, 5.9)].

On the other hand, women who had no complications during their labor and delivery were nearly six times more likely to have the intention to deliver in the health facility in their future pregnancy [AOR = 6.0 (95% CI: 3.5, 10.4)]. This study also revealed that women who received RMC during their current delivery were nearly two times more likely to have the intention to deliver their future pregnancy than those who did not receive RMC [AOD = 1.8 (95% CI: 1.05, 3.10)].

Other independent variables, such as how labor was started, mode of delivery, and place of delivery, did not show significant associations with the outcome (Table 3).

**TABLE 3:** Logistic regression analysis.

Variables	Women’s intention to give birth in the health institution		COR (95% CI)	AOR (95% CI)
	Yes	No		
**Parity**				
Primi parous	258 (96.3)	10 (3.7)	1	1
Multi parous	431 (84.8)	77 (15.2)	0.2 (0.1, 0.4)	0.18(0.08,0.36)*
**Planned pregnancy**				
Yes	613 (90.4)	65 (9.6)	2.7 (1.5, 4.6)	3.1 (1.6, 5.9)*
No	76 (77.6)	22 (22.4)	1	**1**
**Mode of delivery**				
SVD	520 (91.4)	49 (8.6)	2.5 (1.5, 4.2)	1.6 (0,7, 3.5)
Instrumental	50 (84.7)	9 (15.3)	1.3 (0.5, 3.0)	0.8 (0.2, 2.1)
C/S	119 (80.4)	29 (19.5)	1	1
**Complication during labor and delivery**				
Yes	115 (70.6)	48 (29.4)	1	**1**
No	574 (93.6)	39 (6.4)	6.1 (3.8, 9.8)	6.0 (3.5, 10.4)*
**Place of delivery**				
Health center	386 (91.2)	37 (8.8)	1.7 (1.09, 2.7)	0.8 (0.4, 1.6)
Hospital	307 (84.9)	40 (14.1)	1	1
**Received RMC**				
Yes	349	28	2.1 (1.3, 3.4)	1.8 (1.05, 3.10)*
No	340	59	1	1

*Significant variable with *p* ≤ 0.05.

Bold values indicate significantly associated variables.

## Discussion

This study found that women’s intention for institutional delivery service utilization was 88.8% (95% CI: 86.6–91%) of women who intended to use health institutions for the next pregnancy. This finding was lower than that of a study conducted in Addis Ababa (93.2%) (12). The reason for this variation is that women who live in Addis Ababa (the capital city of Ethiopia) may have relatively good knowledge about the importance of having institutional delivery, which could increase their preference to give birth in health institutions. On the other hand, the magnitude of this study was higher compared to the study done in Bahir Dar (66%) (13), Debre Markos (74.3%) (14), Wollaita Soddo Town (75.5%) (15), Debre Tabor town (70.8%) (16), Basra Iraq (83.9%) (17), and rural India (60%) (18). The possible explanation for this difference might be that the above studies were asked about women’s preferences during ANC, which might lower the magnitude because women could change their preferences after they receive delivery services in the health institution depending on how the care is provided for them.

This study also identified associated factors with women’s intention to use health institutions for the next childbirth; the current study showed that multiparous women were 82% less likely to have the intention to give birth in the health institution for their next childbirth. This finding was supported by different studies conducted in Debre Tabor (16), and Northwest Ethiopia (10). Multiparous women might experience different disrespect and abuse that could reduce their intention to use health facilities for their next childbirth (19).

Having a planned pregnancy could increase the likelihood of intention of the woman to give birth in a health facility by 3.1 times. The study performed in Addis Ababa (12) agreed with the current finding. Women who have planned pregnancies prefer to give birth in a health institution because they need and plan to have a healthy baby with safe and clean delivery by using institutional delivery (20).

Women who had not faced complications in health institutions during labor and delivery were six times more likely to use health institutions for the next birth than women who had a complication. In contrast, a study performed in Debre Markos town (14), Jharkhand state, India (21) showed a positive association between complications during labor and delivery and women’s intention to use institutional delivery. This is because women prefer to give birth in health institutions to be free from or have minimum complications during labor and delivery, but if they develop any complications, this might be the reason to regret choosing health institutions for childbirth.

This study showed that women who received RMC during their current labor and delivery had a 1.8 times likelihood of intention to use health institutions for the next childbirth. This finding was supported by a study done in Tanzania (22). This is because RMC is an essential strategy for improving the quality and utilization of maternity care and women’s intention to seek care from health institutions (13).

## Conclusion

This study identified that the magnitude of the intention of the women to give birth in the health institutions was found to be higher but needed intervention for all women to have the intention to use health facilities for childbirth.

According to this study, parity, having planned pregnancy, complications during labor, and delivery, and having RMC were contributing factors to women’s intention to use health institutions for the next childbirth.

## Limitations of the study

■ A causal relationship cannot be established due to the cross-sectional nature of the study.

■ Because of the absence of women who delivered at home, the study cannot show the association between home delivery and intention to use health institutions for childbirth.

## Data availability statement

The raw data supporting the conclusions of this article will be made available by the authors, without undue reservation.

## Ethics statement

The studies involving human participants were reviewed and approved by Debre Berhan University, College of Health Science Research Committee. The patients/participants provided their written informed consent to participate in this study.

## Author contributions

NA and AM designed the study, participated in the data collection, performed the analysis, interpretation of data, and drafted the manuscript. GT and YA designed, approved the proposal with some revisions, participated in data analysis, and revised subsequent drafts of the manuscript. All authors contributed to the article and approved the submitted version.
